# Interaction Effects between Doxorubicin and Hernandezine on the Pharmacokinetics by Liquid Chromatography Coupled with Mass Spectrometry

**DOI:** 10.3390/molecules24193622

**Published:** 2019-10-08

**Authors:** Yang Song, Yuan Zhang, Wei-Peng Zhang, Bao-Zhen Zhang, Ke-Fei Wang, Xue-Song Feng

**Affiliations:** 1 School of Pharmacy, China Medical University, Shenyang 110122, China; songyanglhyb1998@163.com (Y.S.); wpzhang@cmu.edu.cn (W.-P.Z.); piaotaihengmartin@163.com (B.-Z.Z.); wl5712367805@sina.com (K.-F.W.); 2 Department of Pharmacy, National Cancer Center/National Clinical Research Center for Cancer, Chinese Academy of Medical Sciences and Peking Union Medical College, Beijing 100021, China; zhangyuan@cicams.ac.cn

**Keywords:** doxorubicin, hernandezine, LC-MS/MS, pharmacokinetic study, drug–drug interaction

## Abstract

Doxorubicin (DOX) is an effective anti-tumor drug widely used in clinics. Hernandezine (HER), isolated from a Chinese medicinal herb, has a selective inhibitory effect on DOX multidrug resistance, making DOX more effective in treating cancer. The aim of this study was to investigate the effect of the interaction of HER and DOX on pharmacokinetics. Male Sparague–Dawley rats were randomly divided into three groups: a single DOX group, a single HER group, and a combination group. Plasma concentrations of DOX and HER were determined by the LC-MS/MS method at specified time points after administration, and the main pharmacokinetic parameters were estimated. The results showed that there were significant differences in the C_max_ and AUC_0–∞_ of DOX in the single drug group and combined drug group, indicating that HER could improve the absorption of DOX. However, DOX in combination, in turn, reduced the free drug concentration of HER, possibly because DOX enhanced the HER drug–protein binding effect. The results could be used as clinical guidance for DOX and HER to avoid adverse reactions.

## 1. Introduction

Doxorubicin (DOX), as an anti-tumor drug widely used in clinical treatment, has an obvious curative effect on various tumors, including leukemia, malignant lymphoma, and various solid tumors [1,2,3]. However, its long-term or high-dose clinical use often leads to irreversible congestive heart failure, which limits its application [4,5]. In previous studies, the cardiotoxicity may be related to the formation of free radicals, lipid peroxidation, Ca^2+^ overloading, and the activation of apoptotic factors [6,7]. In order to find an effective way to solve this problem, many research studies have been carried out, but the results were not satisfactory [8]. The multidrug resistance (MDR) of DOX is a major obstacle to its application in tumor chemotherapy. Although various mechanisms are known to be involved in MDR phenotypes, the overexpression of some members of the ATP-binding cassette (ABC) protein family is considered to be a major contributor to MDR development in tumor cells [9].

Hernandezine (HER), a dibenzyl isoquinoline alkaloid isolated from traditional Chinese medicine, has long been used in the treatment of hypertension [10,11]. HER has been proved to be able to prevent hair cell aminoglycoside-induced injury [12], inhibit protein kinase C signal events in human peripheral blood T cells [13] and neuronal nicotinic acetylcholine receptors (nAChRs), [14], and block non-voltage-operated Ca^2+^ entry activated by intracellular Ca^2+^ store depletion induced by thapsigargin in rat glioma C6 cells [15] and in human leukemic HL-60 cells [16]. In addition, HER was found to be an effective MDR modulator. Recent studies have shown that HER, as a new AMPK activator, could induce autophagy death in drug-resistant cancers [17]. Furthermore, HER could effectively inhibit the transport function of ABCB1 relative to MDR-linked ABC drug transporters ABCC1 and ABCG2 to enhance the drug-induced apoptosis of tumor cells [18].

In summary, HER has a selective inhibitory effect on the MDR of DOX, which could make DOX better in the treatment of cancer [17,18]. Therefore, it is of great significance to study the pharmacokinetic characteristics of the two drugs in combination. The purpose of this study was to investigate the interaction between HER and DOX on the pharmacokinetics: whether HER could improve the absorption of DOX or the effect in turn, and whether HER could reduce the accumulation of DOX in myocardial tissue.

At present, there are several analytical methods for determining DOX [19,20], but only one article [21] for HER for the pharmacokinetics of rats by LC-MS. According to the previous investigations, the sample preparation methods adopted are mainly focused on a single protein precipitation step. However, there have been no reports of the simultaneous determination of DOX and HER in rat plasma. Therefore, we developed and verified a simple, specific, and sensitive LC-MS/MS method for the simultaneous determination of DOX and HER in rat plasma, and applied it to the pharmacokinetic study of rats to evaluate the effect of HER and DOX interaction on pharmacokinetics.

## 2. Results and Discussion

### 2.1. Method Development

**Figure 1:**
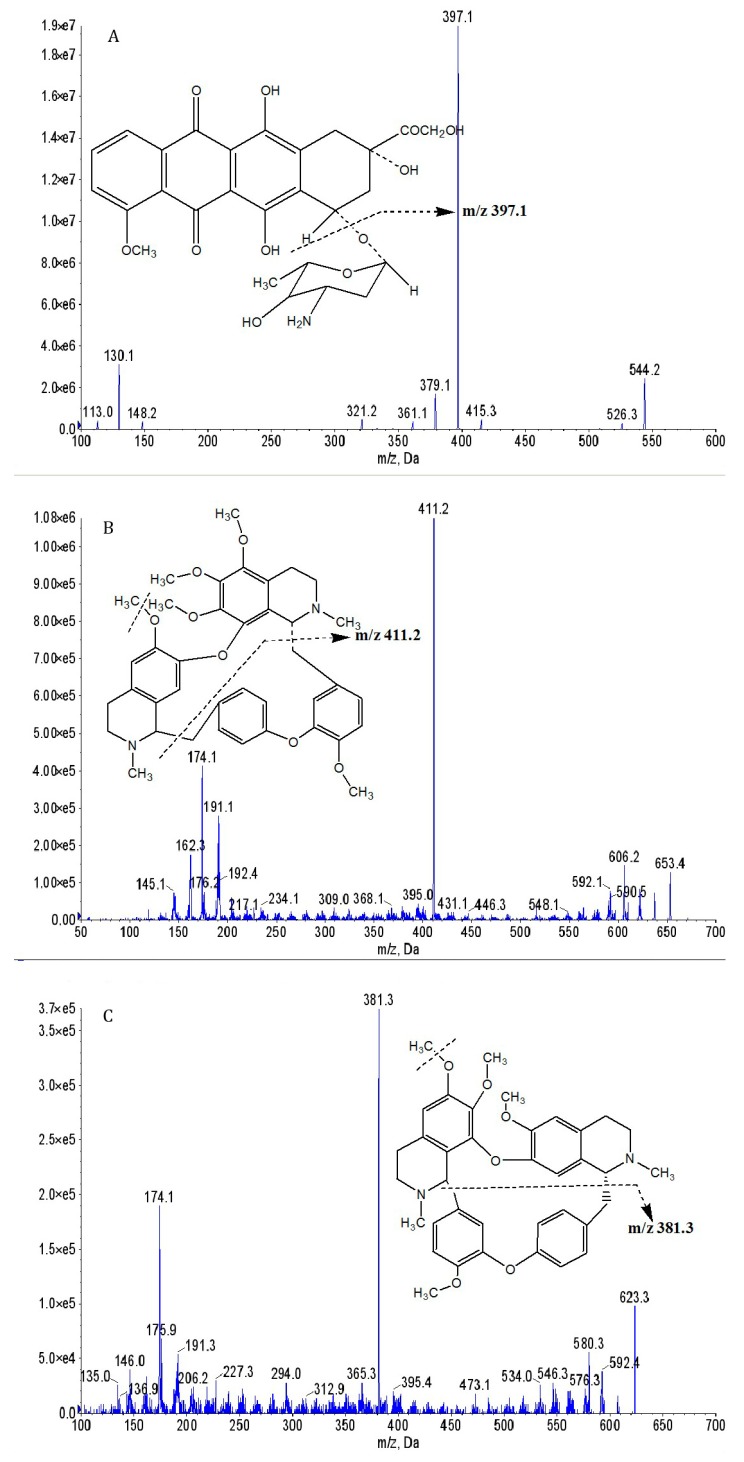
Representative MS of (**A**) doxorubicin (DOX), (**B**) hernandezine (HER), and (**C**) tetrandrine (IS).

To optimize the MS conditions for detecting DOX, HER, and tetrandrine (IS), all the operational parameters were carefully optimized. The analysis showed that positive ion detection has a stronger response than negative ion detection. The MS/MS ion transition was monitored in the MRM mode to improve the specificity and sensitivity of the detection method considering the complexity of biological samples. DOX has the strongest peak at *m*/*z* 544.2→379.1, HER has the strongest peak at *m*/*z* 653.4→411.2, and IS (tetrandrine) has the strongest peak at *m*/*z* 623.3→381.3. The structures of the proposed daughter ions were given by referring to the related articles [19,20,22]. The ion spectra and chemical structures of DOX, HER, and IS are shown in Figure 1.

**Figure 2:**
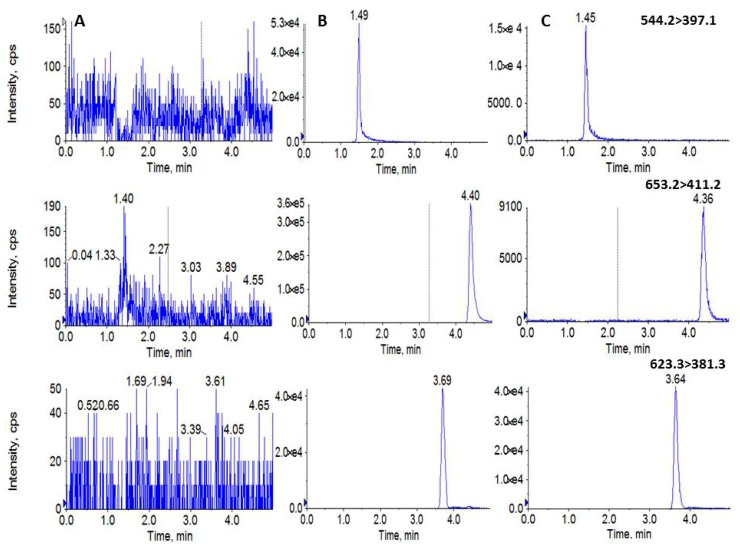
Representative EIC of (**A**) blank plasma; (**B**) blank plasma spiked with doxorubicin, hernandezine at limit of quantification (LOQ) and IS; (**C**) plasma sample after combination administration of the doxorubicin (5 mg/kg) and hernandezine (5 mg/kg) for 2 h.

The chromatographic conditions were optimized, and a good separation effect was obtained with a sharp peak shape, high response, and short run time. The stationary phase and the composition of the mobile phase was studied. An ACQUITY UPLC BEH C_18_ Column (100 mm × 2.1 mm, 1.7 μm) was chosen in this study with good peak symmetry. Different mobile phases (acetonitrile–water and methanol–water or with different concentrations of formic acid or ammonium acetate) were investigated. The results showed that the peak symmetry and response of the acetonitrile–water system were better than those of the methanol–water system. Meanwhile, both gradient elution and isocratic elution were tested, and the result showed that isocratic elution way was more simple, fast, and did not sacrifice any sensitivity and specificity. The retention times of DOX, HER, and IS were 1.46 min, 4.37 min, and 3.65 min, respectively (Figure 2), and the total chromatographic run time was 5.0 min.

In this study, a protein precipitation method was firstly considered to prepare samples, which was simple, accurate, and efficient. The extraction recovery and matrix effect were tested. Different precipitation reagents, such as acetonitrile, methanol, and acetonitrile with 0.1% formic acid, were investigated. The results showed that acetonitrile was the best choice, with a higher extraction rate and lower background interference.

### 2.2. Method Validation

The method validation was conducted in strict accordance with US Food and Drug Administration (FDA) guidelines [23], the content of which consists of selectivity and specificity, linearity, limit of quantification (LOQ), limit of detection (LOD), accuracy, precision, recovery, matrix effect, and stability.

In the process of method development, a selectivity and specificity test was used to verify that the measured substance is the intended analyte to minimize or avoid interference. The selectivity and specificity test of this experiment was demonstrated by the analysis of blank plasma from six individual rats, which was examined by comparing the retention times of DOX, HER, and IS in blank plasma, the addition of DOX and HER at LOQ and IS to blank plasma, and a plasma sample 2 h after an intravenous administration of the mixture of DOX and HER. The blank plasma should be free of interference at the retention times of the analytes and the IS, and which in spiked samples and actual samples should be consistent, respectively. A typical MRM chromatogram of mixed blank plasma in rats, spiked plasma samples with DOX and HER at LOQ and the IS, and plasma samples of rats after an intravenous injection of a mixture of DOX (5 mg/kg) and HER (5 mg/kg) for 2 h is shown in Figure 2. The results showed that there was no significant endogenous interference in the retention time of the analyte under the established chromatographic conditions.

Calibration curves were established by plotting the peak-area ratio (y) between analytes (DOX or HER) and IS against the nominal concentrations. Linearity was evaluated by weighted (1/*x*^2^) least squares linear regression analysis. The correlation coefficient (*r*) should be greater than 0.99, indicating a good linearity. The limit of detection (LOD) is defined as the lowest detectable concentration, judged by the signal-to-noise ratio (SNR) >10. The limit of quantification (LOQ) was defined as the lowest concentration on the calibration curve, which represents the sensitivity of the method and should be lower than the minimum concentration in all the samples. The linear calibration curve was obtained by plotting the peak area ratio (analytes/IS) versus DOX and HER concentration. A weighted (1/*x*) quadratic least-square regression analysis gave typical regression curves. The calibration curves, correlation coefficients, detection ranges, and LOQ of DOX and HER in plasma and myocardial tissues are shown in Table 1. The calibration curves had good linearity with the corresponding range of DOX and HER (r > 0.99). Under the optimized conditions, DOX LOQ was <4.0 ng/mL, and HER LOQ was <2.0 ng/mL in rat plasma, judging from the signal-to-noise ratios (SNR) of >10.

Accuracy and precision tests are critical in determining whether the method is ready for validation, and involve analyzing replicate quality controls (QCs) at different concentrations throughout the assay range. Specifically, the intraday and interday precisions and accuracies were obtained by analyzing five replicates of QC samples at three levels for three consecutive days. Precision, defined as the relative standard deviation (RSD), should be within 15% at each QC level. Accuracy expressed as relative error (RE) must be within ± 15%. Except for the LOQ level, the RSD value of precision should be within 20%, and the RE value of accuracy should be within ± 20%. The intraday and interday precision of the QC samples of DOX and HER were lower than 9.3% and 5.6%, respectively. The accuracy of DOX was −14.0% to 5.5%, and the accuracy of HER was −9.0% to −0.8% (see Table 2). All the assay values were within the range of acceptable variables, indicating that the established method was precise and accurate.

Recovery of the analytes should be optimized to ensure that the extraction is efficient and reproducible. Recoveries of the analytes at three QC levels (*n* = 5) were determined by comparing the peak area ratios of the analytes to IS from QC samples with those of analyte solutions spiked with post-extracted matrix at equivalent concentrations. The matrix effect was examined to assess the possibility of ion suppression or enhancement. The matrix effect was measured by comparing the peak area ratios of the analytes to IS in solutions spiked with the blank processed matrix with the solutions at three QC levels. In common, it was considered that the matrix effect was obvious if the ratio was less than 85% or more than 115%. The recovery and matrix effect data of DOX and HER in rat plasma were shown in Table 3. The matrix effect range of all analytes was 92.9 ± 4.3% to 112.8 ± 1.8%, and the RSD value was lower than 11.6%. The average recovery of DOX and HER at three QC levels was 88.7 ± 6.2% to 108.4 ± 4.9%, and the RSD value was lower than 7.0%. The results showed that this method had no matrix effect, and could be used for biological analysis.

Stability was conducted by analyzing three replicates of the samples at three QC levels under the following conditions, including bench top stability after 4 h of exposure at room temperature, auto-sampler stability after 24 h of storage in the auto-sampler at 4 °C, freeze/thaw stability evaluated for three freeze–thaw cycles after freezing at −80 °C and thawing at room temperature, and long-term stability storage at −80 °C for 30 days. The samples were considered stable if the average percentage concentration deviation (expressed as RSD) was within 15% of the actual value. The stability results are shown in Table 4. The variation of all the stability studies was less than 15.0%, which met the standard of stability measurement. Therefore, this method could be used for routine analysis.

### 2.3. Pharmacokinetics

**Figure 3:**
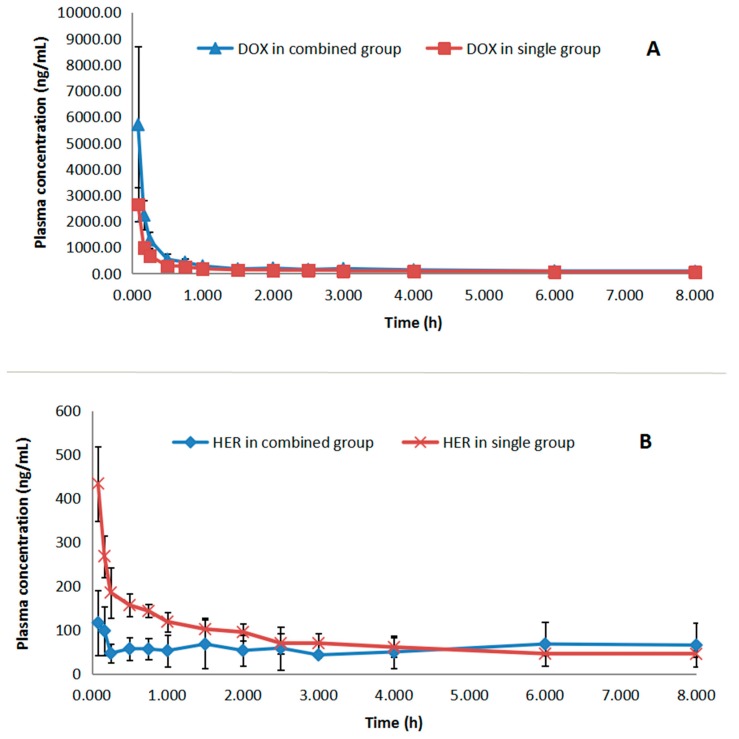
(**A**) Mean plasma concentration–time curves of doxorubicin in a single doxorubicin group and combination group; (**B**) Mean plasma concentration–time curves of hernandezine in a single hernandezine group and combination group.

This validated method has been successfully applied to the determination of plasma concentration of DOX and HER in rats. In this study, we compared the pharmacokinetic parameters of DOX in the combined treatment group with those in the single treatment group. The pharmacokinetic profiles of HER were also compared in the same way. The mean plasma concentration–time profiles of DOX and HER for the three groups were shown in Figure 3. The pharmacokinetic parameters of DOX and HER in rats following the intravenous administration of single DOX (5 mg/kg), single HER (5 mg/kg), and a combination of DOX and HER (5 mg/kg, respectively) were shown in Table 5.

The C_max_ of DOX in the single group and combined group was 2647 ± 650 ng/mL and 5703 ± 2980 ng/mL, respectively. Meanwhile, the AUC_0–∞_ was 1412 ± 114 ng/mL and 2453 ± 218 ng/mL, respectively. Meanwhile, the t_1/2_ was 4.6 ± 0.8 h and 4.2 ± 0.6 h, and the MRT_0–∞_ was 4.9 ± 0.9 h and 4.5 ± 0.5 h, respectively. Significant differences of C_max_ and AUC_0–∞_ of DOX were observed between the single and combined groups with equivalent doses of DOX administration, which indicated that HER could increase the absorption of DOX. However, there was no significant difference between the t_1/2_ and MRT_0–∞_ of DOX, which indicated that HER had no effect on DOX’s elimination and excretion. In turn, we could see from the plasma concentration–time curves of HER in two treatment groups in Figure 3 that the combination use of DOX made the pharmacokinetic behavior of HER no longer fitted to a non-compartmental model that was used to calculate the pharmacokinetic characteristics in this study. However, we were still able to reach a conclusion from the plasma concentration–time curve and the pharmacokinetic characteristic of HER that the free drug concentration of HER was reduced by the combination use of DOX. The possible reason might be the enhancement of DOX on the drug–protein binding of HER.

**Figure 4:**
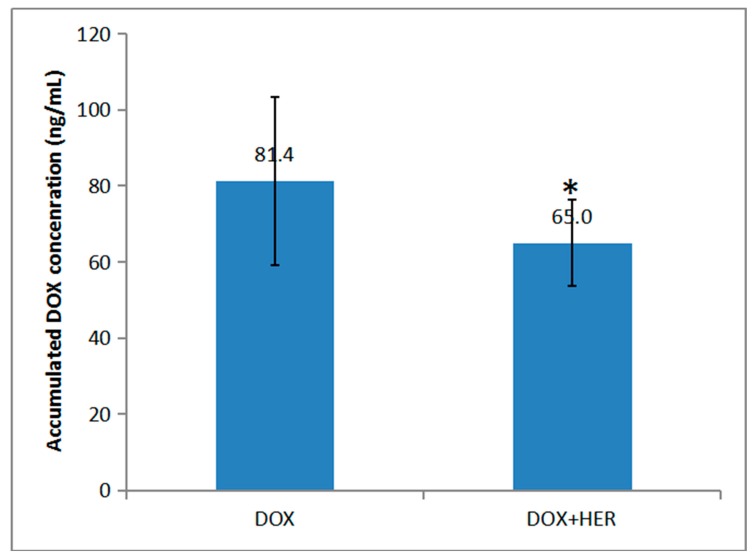
The comparison of the accumulated concentrations of doxorubicin in myocardial tissues 8 h after the intravenous administration of doxorubicin and doxorubicin + hernandezine (mean ± SD, *n* = 6, *p* < 0.05).

The comparison of the accumulated concentrations of DOX in myocardial tissue 8 h after intravenous administration of single DOX and combination of DOX and HER was investigated as shown in Figure 4. A significant difference between the two groups could be observed (*p* < 0.05), indicating that HER was able to reduce the accumulation of DOX in myocardial tissue. Meanwhile, recent studies demonstrated that doxorubicinol (DOX-ol), a secondary alcohol metabolite of DOX [24,25], which may have caused cardiac toxicity by being poorly cleared from the heart and accumulating there to form a long-lived toxicant to heart [26], was to blame. Therefore, the next step is to study whether HER could inhibit the conversion of DOX into DOX-ol, which might be considered as a therapeutic target for DOX-induced cardiac toxicity.

## 3. Experimental

### 3.1. Chemicals and Reagents

DOX (purity over 99%) was obtained from Dalian Meilun Biotech Co., Ltd. (Dalian, China). HER and tetrandrine (purity over 99%) were purchased from Chengdu Biopurity Phytochemicals Ltd. (Chengdu, China). Ammonium acetate, HPLC-grade, was purchased from Dikma Company (Lake Forest, CA 92630, USA). Acetonitrile and methanol, LC-MS-grade, were purchased from Merck KGaA Company (Darmstadt, Germany). Ultra-pure water was provied using a Millipore Milli-Q system (Millipore, Bedford, MA, USA). Other chemical reagents were of analytical grade.

### 3.2. Animals

Sprague–Dawley rats (male, 250 ± 20 g) were supplied by the Experimental Animal Research Center, China Medical University, China. The rats were raised in a temperature-controlled room at 24 ± 2 °C for free feeding and water intake, and the light/dark cycle was 12 h. The rats were fed for 2 weeks to adapt to the laboratory environment. All the rats fasted for 12 h before the experiment, but with water supplied freely. The protocol for animal care and use in our study (protocol number # CMU2019194) was approved by the Institutional Animal Care and Use Committee at China Medical University.

Eighteen rats were randomly divided into three groups (six rats in each group) and given intravenous treatment with different drugs: group A, DOX (5 mg/kg); group B, HER (5 mg/kg); group C, DOX + HER (5.0 mg/kg, respectively). The injection was prepared in normal saline with 0.5% *v/v* DMSO.

### 3.3. Instrumentation and Conditions

The biological samples were analyzed with an Agilent series 1290 UHPLC system (Agilent Technologies, Santa Clara, CA, USA), which was coupled to an AB 3500 triple quadrupole mass spectrometer (AB Sciex, Ontario, ON, Canada) with an electrospray ionization (ESI) source. Data acquisition and instrument control were performed using the 1.6.3 version Analyst software package (AB Sciex, ON, Canada).

The separation process was performed on an ACQUITY UPLC BEH C_18_ Column (100 mm × 2.1 mm, 1.7 μm, Agilent Technologies, Santa Clara, CA, USA). The column temperature was set at 40 °C. The mobile phase was composed of acetonitrile and 10 mM ammonium acetate aqueous solution (70:30, *v*/*v*) at the flow rate of 0.3 mL/min in an isocratic elution manner. The injection volume was set at 10 μL.

DOX and HER were quantitatively determined with MRM in the positive ion mode. The MS condition was as follows: the ion spray voltage (IS) was set at 5500 V, the turbo spray temperature (TEM) was set at 500 °C, and the nebulizer gas and heater gas were set at 50 and 50 arbitrary units, respectively. The curtain gas (CUR) was kept at 40 arbitrary units, and the interface heater was on. The collision cell exit potential (CXP) and entrance potential (EP) were set at 7.0 V and 10.0 V, respectively. The declustering potentials (DPs) of DOX, HER, and IS were set at 160 V, 218 V, and 87 V; the collision energies (CEs) were 60 eV, 60 eV, and 27 eV, respectively. Nitrogen was used in all cases. The optimization of the MS transitions for quantification were accomplished as DOX *m*/*z* 544.2→379.1, HER *m*/*z* 653.4→411.2, and IS (tetrandrine) *m*/*z* 623.3→381.3, respectively. Moreover, the qualifier ions for DOX, HER, and IS were set at *m*/*z* 321.1, *m*/*z* 191.1, and *m*/*z* 174.1, respectively.

### 3.4. Preparation of Stock Solutions, Working Solutions, Calibration Standards, and Quality Control Samples

The standard substances of the analytes were accurately weighed and dissolved in methanol to prepare the DOX and HER stock solution with the concentration of 1.0 mg/mL, respectively. The working solution for preparing calibration standards and QC samples was obtained by diluting the stock solution with acetonitrile–water (50:50, *v*/*v*). The IS working solution with a concentration of 200 ng/mL was also prepared. The stock solution and working solution were placed under 4 °C dark condition and brought to room temperature before use.

The calibration standards were prepared by spiking 50 μL of rat blank plasma (or blank myocardial tissue homogenate) with 20 μL of the working solution. The concentration of DOX in rat plasma and myocardial tissue homogenate ranged from 32 to 8000 ng/mL, and HER ranged from 20 to 4000 ng/mL. Low, medium, and high quality control (QC) samples were prepared in the same way as above (40.0, 400, and 3200 ng/mL for DOX; 80.0, 800, and 4000 ng/mL for HER) in both rat plasma and myocardial tissue homogenate. Each concentration needed three replicates.

### 3.5. Sample Preparation

In this study, DOX, HER, and IS were extracted from the biological matrix (plasma and myocardial tissue homogenate) by routine step protein precipitation. Detailed steps were as follows: take 50 µL of biological matrix, add 20 µL of acetonitrile–water (50:50, *v*/*v*), and 20 µL of the IS solution, and add 200 µL of precipitation reagent acetonitrile, placed in a 1.5-mL EP tube. Vortex for 1 min, followed by centrifuging at 14,000 rpm for 10 min. Transfer 200 µL of supernatant to another clean 1.5-mL EP tube and centrifuge at 14,000 rpm for another 3 min. Then, an aliquot of 10 µL of the supernatant was injected into the LC-MS system for analysis.

### 3.6. Pharmacokinetic Study

The method was used to determine the concentration–time profiles of DOX and HER in the plasma of rats after the intravenous administration of DOX (5.0 mg/kg), HER (5.0 mg/kg), and the mixture of DOX and HER (5.0 mg/kg, respectively). Blood samples (250 μL) were taken from the orbital vein at 5 min, 10 min, 15 min, 30 min, 45 min, 1 h, 1.5 h, 2 h, 2.5 h, 3 h, 4 h, 6 h, and 8 h, respectively, and were injected into heparinized 1.5-mL EP tubes. Heparin (2 mg/mL blood volume) was used as an anticoagulant for this study, and blood samples were immediately centrifuged at 14,000 rpm for 10 min at room temperature, followed by a supernatant plasma layer collected and stored at −80 °C for analysis.

After the last blood sample was taken, the rats were sacrificed for cervical dislocation. The heart was removed and rinsed with cold saline to remove the superficial blood. Then, it was blotted dry with filter paper and weighed accurately. After that, the heart was homogenized with normal saline to prepare a homogenate (0.2 g/mL). All samples were stored at −80 °C for analysis.

Plasma concentration–time plots were plotted, and the PK parameters were evaluated by means of non-compartmental pharmacokinetic analysis using DAS 3.2.8 pharmacokinetic program [27]. The PK parameters concerned include half-life (t_1/2_), mean residence time (MRT), area under the plasma concentration–time curve (AUC), clearance (CL), etc. Data was expressed as mean ± SD. The pharmacokinetic parameters were compared using Student’s t-test. Differences were considered to be significant at a level of *p* < 0.05.

## 4. Conclusions

An LC-MS method for the simultaneous determination of DOX and HER in rat plasma was established. The method is sensitive, accurate, easy to follow, and suitable for the pharmacokinetic study. This analytical method has been successfully applied to the pharmacokinetic study of DOX and HER in rats.

The results of this study showed that there were significant differences in the pharmacokinetic parameters of DOX and HER after the intravenous administration of a single dose of DOX, single dose of HER, and a combination of the two. This result might help to explain the influence of DOX and HER interaction on pharmacokinetics and provide a basis for guiding clinical medication.

## Figures and Tables

**Table 1:** Calibration curves of doxorubicin and hernandezine in plasma and myocardial tissue homogenate of rats.

Analytes	Samples	Calibration Curves	Correlation Coefficients (r)	Linear Ranges (ng/mL)	LOQs (ng/mL)
DOX	Plasma	Y = 0.0047 + 0.00015x	0.994	32–8000	32
	Heart	Y = −0.0028 + 0.00019x	0.992	32–8000	32
HER	Plasma	Y = −0.0038 + 0.00298x	0.998	20–4000	20
	Heart	Y = −0.0197 + 0.00373x	0.994	20–4000	20

**Table 2:** Precision and accuracy of doxorubicin and hernandezine in plasma of rats (n = 5). RSD: relative standard deviation.

Analytes	QC Conc. (ng/mL)	Intraday		Interday	
		Precision (RSD, %)	Accurary (mean %)	Precision (RSD, %)	Accurary (mean %)
DOX	80	1.6	−5.7	4.3	−6.0
	800	6.4	5.5	6.8	5.3
	4000	4.0	−14.0	9.3	−3.6
HER	40	5.6	−5.2	4.6	−3.3
	400	5.6	−0.8	3.1	−1.4
	3200	1.4	−7.8	1.9	−9.0

**Table 3:** Matrix effect and recovery of doxorubicin and hernandezine in plasma of rats (n = 5).

Analytes	QC Conc. (ng/mL)	Matrix Effect		Recovery	
		Mean ± SD (%)	RSD (%)	Mean ± SD (%)	RSD (%)
DOX	80	112.8 ± 1.8	1.6	88.7 ± 6.2	7.0
	800	95.3 ± 11.1	11.6	103.2 ± 2.6	2.5
	4000	92.9 ± 4.3	4.6	95.1 ± 2.1	2.2
HER	40	104.0 ± 1.7	1.7	91.7 ± 4.3	4.7
	400	94.2 ± 1.5	1.6	108.4 ± 4.9	4.6
	3200	94.5 ± 1.7	1.8	93.2 ± 0.6	0.6

**Table 4:** Stability results for doxorubicin and hernandezine in plasma of rats under different storage conditions (n = 3).

Analytes	QC Conc. (ng/mL)	Bench Top Stability (at Room Temperature for 4 h)	Auto-Sampler Stability (at 4 °C for 24 h)	Freeze/Thraw Stability	Long Term Stability (at −80 °C for 30 days)
DOX	80	1.6	4.3	3.4	3.1
	800	6.4	6.8	6.1	9.6
	4000	4.0	0.2	8.2	5.0
HER	40	5.5	4.7	4.1	1.7
	400	5.5	3.1	1.6	0.8
	3200	1.3	1.9	1.9	1.6

**Table 5:** Non-compartmental pharmacokinetic parameters of hernandezine and doxorubicin in a single doxorubicin group, single hernandezine group, and combination group (n = 6).

Pharmacokinetic Parameters	Single DOX Group	Single HER Group	Combination Group	
			DOX	HER
***C_max_* (ng/mL)**	2647 ± 650	433.6 ± 85.2	5703 ± 2980	116.6 ± 74.0
***T_max_* (h)**	0.083	0.083	0.083	0.083
***Ke* (1/min)**	0.150 ± 0.03	0.090 ± 0.01	0.164 ± 0.02	-
***t*_1/2_ (h)**	4.6 ± 0.8	7.7 ± 1.2	4.2 ± 0.6	-
**AUC_0–*t*_ (ng h/mL)**	1109 ± 102	647.2 ± 54.9	1965 ± 142.5	49.9 ± 12.5
**AUC_0–∞_ (ng h/mL)**	1412 ± 114	1154 ± 85	2453 ± 218	-
**MRT_0–∞_ (h)**	4.9 ± 0.9	10.1 ± 1.4	4.5 ± 0.5	-
***CL*/*F* (L/kg/h)**	3.5 ± 0.5	4.3 ± 0.5	2.0 ± 0.2	-
***Vd*/*F* (L/kg)**	23.6 ± 7.1	48.3 ± 5.6	12.4 ± 2.1	81.8 ± 2.3
